# CD133 Targeted PVP/PMMA Microparticle Incorporating Levamisole for the Treatment of Ovarian Cancer

**DOI:** 10.3390/polym12020479

**Published:** 2020-02-20

**Authors:** Yu-Chi Wang, Meng-Yi Bai, Ying-Ting Yeh, Sung-Ling Tang, Mu-Hsien Yu

**Affiliations:** 1 Department of Obstetric and Gynecology, Tri-Service General Hospital, National Defense Medical Center, Taipei 10607, Taiwan; yuchitsgh@gmail.com (Y.-C.W.); hsienhui@ms15.hinet.net (M.-H.Y.); 2 Graduate Institute of Biomedical Engineering, National Taiwan University of Science and Technology, Taipei 10607, Taiwan; yyt.yeh@gmail.com; 3 Biomedical Engineering Program, Graduate Institute of Applied Science and Technology, National Taiwan University of Science and Technology, Taipei 10607, Taiwan; 4 Adjunct Appointment to the Department of Biomedical Engineering, National Defense Medical Center, Taipei 10607, Taiwan; 5 Department of Pharmacy Practice, Tri-Service General Hospital, Taipei 10607, Taiwan; tasuling@gmail.com

**Keywords:** levamisole, drug carrier, microparticle, ovarian cancer, electrospray

## Abstract

Levamisole (LEVA) is used to treat worm infections, but it can also inhibit cancer cell growth by inhibiting the aldehyde dehydrogenase pathway. Therefore, here, we developed a drug carrier targeting CD133, a biomarker overexpressed in ovarian cancer cells. The particle structure and cytotoxicity of the prepared LEVA-containing particles—called LEVA/PVP/PMMA microparticles (MPs) (because it used matrix material polyvinylpyrrolidone (PVP) and poly(methylmethacrylate) (PMMA))—were investigated in the ovarian cancer cell lines SKOV-3 and CP70. The particle size of the MPs was determined to be 1.0–1.5 µm and to be monodispersed. The hydrophilic property of PVP created a porous MP surface after the MPs were soaked in water for 20 min, which aided the leaching of the hydrophilic LEVA out of the MPs. The encapsulation efficiency of LEVA/PVP/PMMA MPs could reach up to 20%. Free-form LEVA released 50% of drugs in <1 h and 90% of drugs in 1 day, whereas the drug release rate of LEVA/PVP/PMMA MPs was much slower; 50% released in 4 h and only 70% of drugs released in 1 day. In the in vitro cell model test, 5 mM free-form LEVA and 0.1 g/mL CD133 targeted LEVA/PVP/PMMA MPs reduced SKOV-3 cell viability by 60%; 0.1 g/mL LEVA/PVP/PMMA MPs was equivalent to a similar dosage of the free drug. In addition, the cytotoxicity of CD133-conjugated LEVA/PVP/PMMA MPs shows a different cytotoxicity response toward cell lines. For SKOV-3 cells, treatment with free-form LEVA or CD133-conjugated LEVA/PVP/PMMA MPs exerted dose-dependent cytotoxic effects on SKOV-3 cell viability. However, CD133-conjugated LEVA/PVP/PMMA MPs demonstrated no significant dose-dependent cytotoxic efficacy toward CP70 cells.

## 1. Introduction

Ovarian cancer is the leading cause of death in women because of its high recurrence rate and eventual resistance to cytotoxic chemotherapy [1,2]. Chemotherapeutic drugs are frequently used to treat many cancers after surgery to prevent cancer cell metastasis; however, most cancer cells develop inherently or acquire resistance after exposure to chemotherapeutic treatment, highlighting the necessity for novel therapeutic approaches to overcome chemoresistance and improve treatment outcome.

Several nanoparticles (NPs) have been extensively developed to overcome drug resistance. The NP-based drug delivery system is highly effective in cancer treatment because of its specific penetration ability, intracellular delivery, and feature of interacting with a high surface area of cells. Therefore, NPs increase the concentration of therapeutic payloads at the disease site, which minimizes the side effects of the therapeutic drug [3,4,5,6,7]. However, the use of nanoparticles is not always a must criterion, which depends on the way in which they are applied or intended use. For example, some researchers reported the use of microparticles for using in drug delivery [8,9], like encapsulation of enzymes for gastrointestinal delivery, implantation for sustained release and for embolization chemotherapy.

Cancer stem cells (CSCs) have two crucial traits; they promote tumor initiation and self-renewal. CSCs were identified in 1997 by Dick and Bonnet [10], who reported that CSCs can proliferate the cell population in human myelomonocytic acute myeloid leukemia. CSCs are more resistant to chemotherapy and radiation than non-CSCs. Several CSC markers have been identified in various carcinomas, including lung, colon, breast, prostate, liver, pancreatic, and head and neck squamous cell carcinomas [11,12,13,14]. CD133 is a membrane-bound pentaspan glycoprotein, first identified in neuroepithelial stem cells in mice and later in human tissue [15]. The current physiologic role of this receptor is in primitive cell differentiation and epidermal–mesenchymal interaction. CD133 is apparently associated with WNT signaling pathway and thus cell proliferation and seems to be negatively associated with patient survival [16,17,18,19,20,21]. CD133 overexpression in tumors remains poorly understood. Here, we study the routes of metastasis of tumor cells mainly through the lymphatic or blood vessel system. Therefore, hydrophilic or hydrophobic property is a crucial factor in enhancing particle uptake by the lymphatic or reticuloendothelial system to onsite drug delivery and simultaneously killing metastatic cancer cells. Therefore, it is necessary to strike a balance between the hydrophobicity of encapsulation matrix and the encapsulated drug. We thus developed a poly(methylmethacrylate) (PMMA)-polyvinylpyrrolidone (PVP) NP that conjugated with CD133 and levamisole (LEVA) to fight against the ovarian cancer cell line by using the bioconjugation method. Normally, ovarian cancer predominantly transfers through the pelvic lymph nodes. Compared to the vascular system, the lymphatic system is more favorable for hydrophobic and micro particles to facilitate the uptake by the lymphocyte [22]. Therefore, we use highly hydrophobic material, PMMA, as one of the main constituents of particles. Moreover, we enlarged the particle size to increase the recognition and uptake by lymphocytes. Thus, the particles can selectively eliminate cancer cells in the lymphatic system and apparently block the cancer metastasis from the ovary. LEVA has shown its potential as an adjuvant treatment on colon cancer [22,23], but it was withdrawn from market because of its serious adverse effects, including agranulocytosis. In this study, LEVA was encapsulated in PMMA and PVP carriers to be candidates for intrauterine drug delivery via cervix injection. Therefore, another constituent, i.e., PVP, was introduced to the encapsulation matrix to enable the water erosion of the particle and the following release of LEVA. To the best of our knowledge, no studies have reported using PMMA and PVP as materials with encapsulation of LEVA for drug delivery through uterine.

This approach provides a new combination of chemotherapy and a new drug carrier to overcome chemoresistance in ovarian cancer, especially toward those possible metastatic cancer cells in the lymphatic system [24].

## 2. Materials and Methods

### 2.1. Materials

Levamisole (LEVA, molecular weight: 204 g/mole, Sigma-Aldrich, St. Louis, MO, USA), PVP (molecular weight: 55,000 g/mole, Sigma-Aldrich, MO, USA), PMMA (molecular weight: 120,000 g/mole, Sigma-Aldrich, MO, USA), and polyethylenimine (branched, molecular weight: 10,000 g/mole, Sigma-Aldrich, MO, USA) were obtained from Sigma-Aldrich (MO, USA). Roswell Park Memorial Institute-1640 (RPMI-1640), antibiotic solution (10,000 IU/mL penicillin and 10 mg/mL streptomycin), fetal bovine serum, and phosphate-buffered saline (PBS; pH 7.4) were obtained from Biological Industries (CT, USA). Acetone was purchased from Echo Chemical (Miaoli, Taiwan), and *N*,*N*-Dimethylformamide from Tedia (OH, USA). All other chemicals were of reagent or tissue culture grade and received for direct use without any purification.

### 2.2. LEVA/PVP/PMMA Microparticle Preparation

We set up an electrospray (ES) system as described in our previous studies [25,26,27,28,29]. First, 0.078–2.5 mg LEVA was dissolved in 2 mL of cosolvent [acetone/dimethylformamide (Ace/DMF) = 1/2, v/v] through ultrasonication for 10 min to generate LEVA stock solution. Subsequently, 110 mg of PMMA and 110 mg of PVP were added and completely dissolved in the LEVA stock solution through ultrasonication and magnetic stirring at 30 °C–40 °C, to generate LEVA/PVP/PMMA stock solution, which was delivered using a syringe pump apparatus (NE-300 Just Infusion; New Era Pump Systems, Farmingdale, NY, USA). We used a 20-gauge flat-tipped needle as the capillary tube in this experiment. A positive voltage was applied to the spray nozzle from a direct current high-voltage power supply (Bertan Model 205B-20R; Spellman High Voltage Electronics, Hauppauge, NY, USA) to establish a spray electrical field between the capillary nozzle and the electrically grounded collection substrate. The collection substrate in this setup could be changed between a metallic plate or 2% polyvinyl alcohol (PVA) depending on the purpose of the application. The capillary nozzle was placed at a working distance of approximately 7.5–13.5 cm from the collection substrate. The applied voltage ranged from 7 to 12 kV. The ES modes of the system were monitored by viewing the liquid meniscus at the exit of the capillary nozzle. In addition, the meniscus was illuminated with diffuse light from an optical cable light, and its droplet shape was observed using a microscopic system comprising of a microscopic lens (model 30-43-10, Optem RetroZoom 65; Qioptiq, Rhyl, UK), digital camera (model STC-620PWT; Sentech, Carrollton, TX, USA), and high-resolution liquid crystal display monitor. Finally, the obtained LEVA/PVP/PMMA microparticles (MPs) were collected by decanting the supernatant after centrifugation. Then, the microparticles were quickly washed by distilled water, and the supernatant was removed after centrifugation was carried out three times. These purified LEVA/PVP/PMMA MPs were used for subsequent characterization and assaying. The amount of total free LEVA was next determined through spectrophotometry on a SPECTROstar Nano instrument (BMG LABTECH, Ortenberg, Germany).

### 2.3. CD133-Conjugated LEVA/PVP/PMMA MP Preparation

CD133- LEVA/PVP/PMMA MPs were produced through the conjugation of the CD133 antibody to the aforementioned LEVA/PVP/PMMA MPs by using the surface modification and bioconjugation technique. First, the LEVA/PVP/PMMA MPs were treated with 1 mL of 0.5 N NaOH aqueous solution, heated at 55 °C for 30 min, to generate the carboxylate ion group on the particle surface. Subsequently, these hydrolyzed LEVA/PVP/PMMA MPs were mixed with 1 mL of 0.2% polyethylenimine (PEI) aqueous solution and held steady for 1 h of reaction. After a determined period, the resultant particles were separated from the reaction mixture by centrifugation at 3000 rpm and then washed with deionized water three times to remove unconjugated PEI reactant. These terminal-amine conjugated LEVA/PVP/PMMA MPs subsequently reacted with glutaraldehyde solution (1% w/v) for 30 min and then precipitated down through centrifugation at 3000 rpm. Finally, CD133 antibodies were added to react with the surface-modified LEVA/PVP/PMMA MPs to undergo the bioconjugation reaction for 24 h at 4 °C.

### 2.4. LEVA/PVP/PMMA MP Surface Morphology and Structural Characterization

MP morphology was examined through scanning electron microscopy (SEM) under an accelerating voltage of 20 kV (JSM-6390L; JEOL, Tokyo, Japan). To prevent charge accumulation, all SEM sample specimens were coated with a thin additional layer of platinum film (sputtering time, 90 s). LEVA concentration in LEVA/PVP/PMMA MP suspensions was determined using a spectrophotometer (SPECTRO star Nano). First, the full absorbance spectra of LEVA in the range of UV–vis light were recorded at scanning wavelengths of 200–800 nm, to find that LEVA dissolved in acetonitrile shows the maximum absorption wavelength of 230 nm. A calibration curve was established by plotting the absorption value of LEVA at 230 nm against a series of predetermined concentrations of standard solutions. The encapsulation efficiency was calculated to be approximately 20% (31 μg/mL) and drug loading capacity 0.1%.

### 2.5. In vitro Release Test

A dialysis method using a molecular porous membrane was employed to enable the free LEVA released from the MPs to pass freely through its pores. The dialysis tube with molecular weight cut-off falling from 0.5–1 kD was used (Spectra/Por^®®^ Dialysis Tubing by Spectrum^®®^ Laboratories Inc., NJ, USA). The LEVA/PVP/PMMA MP suspension and free-form LEVA solution (2.5 mg/mL) were poured into dialysis bags, then placed in beakers filled with 250 mL of distilled water. The suspensions were then dialyzed at 37 °C with agitation at 40 rpm. The temperature of the experimental system was set at 37 °C. After a predetermined time interval, all distilled water in the beakers was removed and replaced with 250 mL of fresh distilled water. The LEVA concentration in the used distilled water was subsequently measured based on its absorbance at 230 nm using high performance liquid chromatography (HPLC). Finally, the cumulative percentage of drug release at a specific time interval was calculated according to the total amount of LEVA loading measured at the beginning of this study.

### 2.6. In Vitro Cell Viability or Cytotoxicity Studies toward SKOV-3 and CP70 Cells

A tetrazolium salt 3-(4,5-dimethylthiazol-2-yl)-2,5-diphenyltetrazolium bromide (MTT) assay was performed to assess SKOV-3 and CP70 cell viability after they had been treated with the LEVA/PVP/PMMA MPs suspension. In the typical procedure, a fresh CD133-conjugated LEVA/PVP/PMMA microparticles’ suspension solution was prepared and was subject to the determination of Leva concentration. After determination of concentration, this suspension was used as the stock solution for preparing a series of dilute concentrations to treat cells immediately. SKOV-3 or CP70 cells were seeded onto a 96-well plate at a density of 10^3^–10^4^ cells/100 μL (100 μL/well) in serum-containing RPMI-1640 medium for 12 h at 37 °C in a 5% CO_2_ atmosphere. After 12 h of culture, the medium was removed, and 100 μL of 0.1, 0.25, and 0.5 mM CD133-conjugated LEVA/PVP/PMMA MP suspensions were added to each well for drug treatment. After another 72 h of incubation at 37 °C in a 5% CO_2_ atmosphere, the supernatant was removed from each well and the cells were washed twice with 200 μL of 1 × PBS solution. Finally, 100 μL of MTT reagent was added to each well and the plate was subsequently incubated for 2 h until purple precipitate was visible. Two hours later, all supernatants were discarded and replaced with 200 μL of dimethyl sulfoxide solution. The plate was covered and left in the dark for 10 min at 37 °C in a 5% CO_2_ atmosphere. Spectrophotometry was used to measure the optical density (OD) of the dimethyl sulfoxide extract solution at 570 nm. Subsequently, cell viability was calculated as the ratio of the recorded OD values by using the following equation:

Cell viability = OD of the drug-treated group/OD of the medium-only group × 100%(1)

### 2.7. Surface Morphology and Chemical Composition of Pure LEVA/PVP/PMMA or CD133-Conjugated LEVA/PVP/PMMA Microparticles Using Scanning Electron Microscopy (SEM) and Fourier Transformed-Infrared (FT-IR) Spectroscopy

A scanning electron microscope (SEM) operated at an accelerating voltage of 20 kV (JSM-6390L, JEOL), and equipped with energy dispersive X-ray accessory, was used to characterize the morphology and elemental composition of the produced microparticles. To enhance the charge dissipation, each sample was coated with a thin layer of Pt film using a sputtering coater. The attenuated total reflectance infrared (ATR-IR) spectra of the microparticles were acquired using a FTIR spectrometer that was equipped with an ATR accessory. All spectra were acquired from 12 cycles of accumulation to provide a favorable S/N ratio.

### 2.8. Statistical Analyses

All statistical analyses were performed using IBM SPSS Statistics (IBM, Armonk, NY, USA). All study data are presented as means ± standard deviations. Particularly, one-way analysis of variance, Student’s t-test, and Wilcoxon statistics were used to assess the differences between the control and experimental groups. Differences with *p* values of less than 0.05 were considered statistically significant.

## 3. Results and Discussion

### 3.1. MP Surface Morphological and Structural Characterization Using Field-Emission SEM and TEM

**Figure 1:**
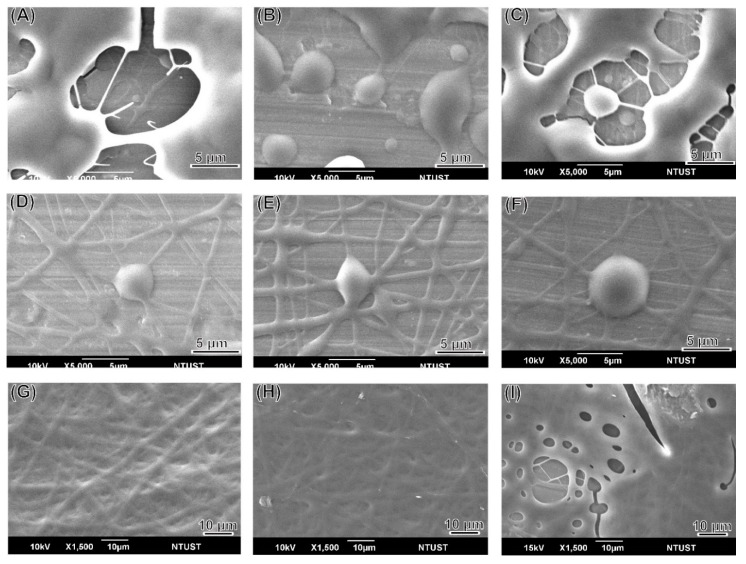
SEM images of the blank polyvinylpyrrolidone (PVP)/poly(methylmethacrylate) (PMMA) microparticle produced from (**A**–**C**) in the Ace/DMF = 1/2 cosolvent system; (**D**–**F**) in the Ace/DMF = 1/1 cosolvent system; (**G**–**I**) in the Ace/DMF = 2/1 cosolvent system.

**Figure 2:**
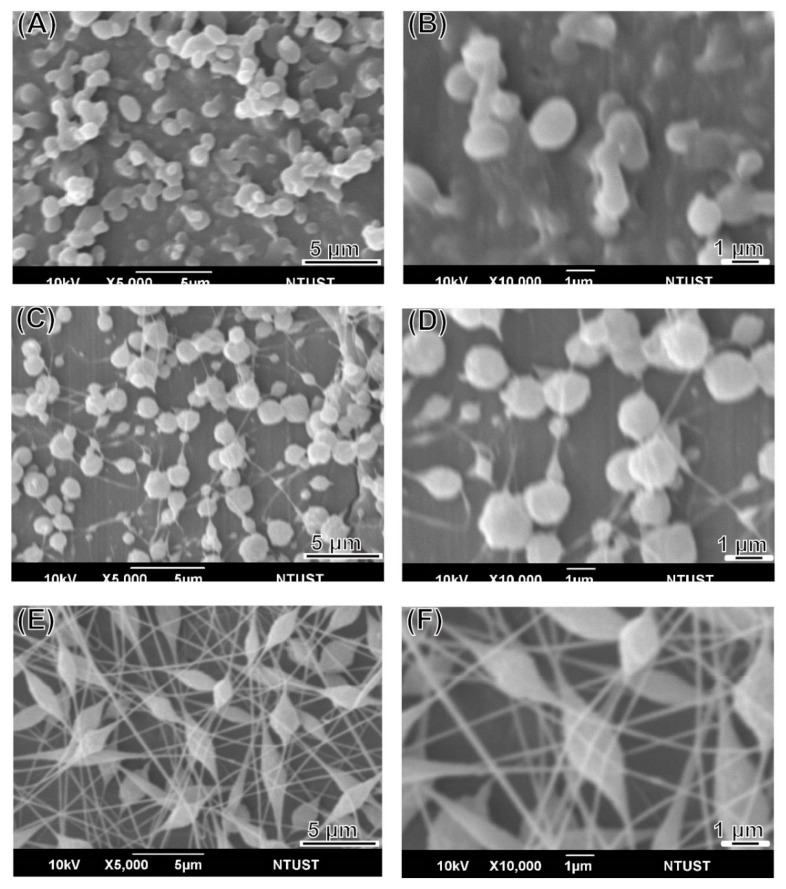
SEM images of particles produced from a series of 10 wt% stock solutions containing the different ratio of PVP and PMMA: (**A**,**B**) 8:2, (**C**,**D**) 5:5 and (**E**,**F**) 2:8 (PVP:PMMA; w/w).

**Figure 3:**
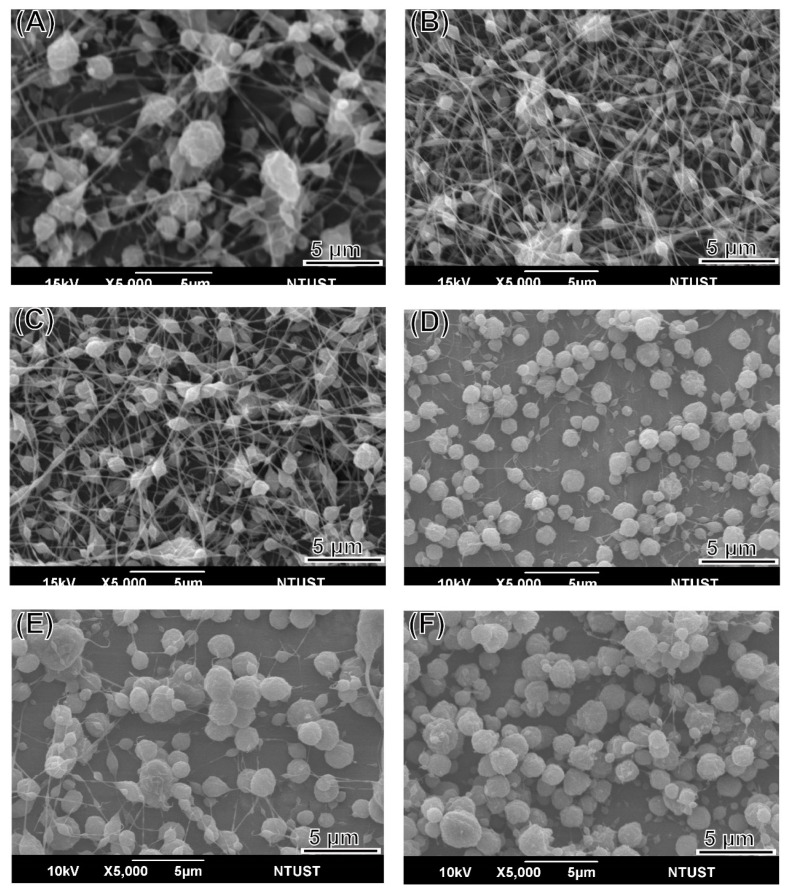
SEM images of levamisole (LEVA)/PVP/PMMA microparticles produced from a series of different concentrations of LEVA: (**A**) 1250 µg/mL, (**B**) 625 µg/mL, (**C**) 312.5 µg/mL, (**D**) 156.25 µg/mL, (**E**) 78.125 µg/mL, (**F**) 39.0625 µg/mL.

LEVA/PVP/PMMA MP morphology was observed in SEM images. All MPs produced were ranging from submicrometers to nanometers, depending on the theory that different solvent systems resulted in different dielectric constants of the stock solution. As shown in Figure 1, the particle numbers produced in the Ace/DMF cosolvent (Ace/DMF = 1/2) system (Figure 1A–C) were increasing more than that in the Ace/DMF cosolvent system (Ace/DMF = 1/1; Figure 1D–F) and Ace/DMF cosolvent system (Ace/DMF = 2/1; Figure 1G–I). This result demonstrates that particle morphology can be significantly influenced by the solvent system. An ES technique was implemented in the particle preparation step. Although solvents with high volatility and low viscosity features can significantly decrease the particle size, our study demonstrated that solubility effects are more decisive than the volatility of the solvent system. The boiling points of Ace and DMF are 56 °C and 153 °C. PMMA can be dissolved in Ace or DMF, but PVP can be dissolved only in Ace. Therefore, a cosolvent system is required for the ES stock solution. However, a balance was necessary between volatility and solubility. We observed that when the volume percentage of Ace in cosolvent increased too much (i.e., Ace/DMF = 2/1), fiber or film products were formed rather than particles. Therefore, we used an Ace/DMF cosolvent system (Ace/DMF = 1/2) for particle preparation. Particle sizes can also be strongly affected by the composition proportion. For example, Figure 2 shows the particle size produced from a series of stock solutions containing different ratios of PVP and PMMA. When the concentration of PMMA was <50%, the morphology of the particles tended to be quasi-spherical. This was possibly because the PMMA used in this study had higher molecular weight (compared with that of PVP), which could dramatically increase the viscosity of the stock solution. Statistical results showed that the distribution of particle sizes was falling in the range of 0.9–1.3 µm, and the best composition ratio of PVP and PMMA to reach a balance between viscosity and solubility was 50:50. Subsequently, another important ingredient, LEVA, was introduced into the aforementioned matrix material. When LEVA concentration increased, the resultant products were fibers rather than particles (as shown in Figure 3) because the ionic form of LEVA increased the viscosity of the stock solution. Thus, the use of the Ace/DMF cosolvent at a 1:2 ratio was appropriate for combination of PMMA and PVA, and relatively low concentration of LEVA (156.25 µg/mL) could strike a balance of viscosity, volatility, and conductivity to generate the homogeneous and spherical shape of LEVA/PVP/PMMA MPs.

### 3.2. In Vitro Release Profile

**Figure 4:**
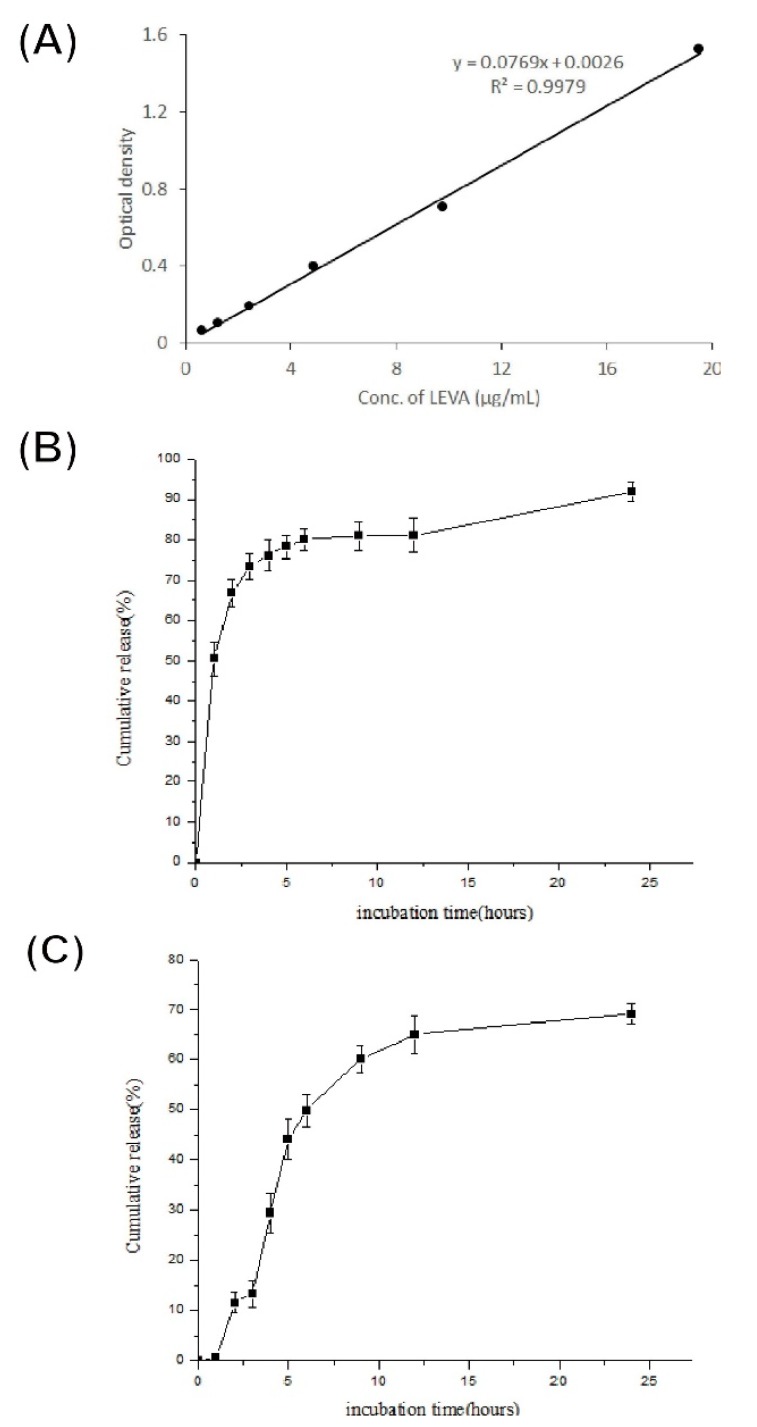
UV–vis spectrum of (**A**) a standard curve constructed from a series of LEVA solutions with known concentrations; (**B**) in vitro cumulative release profile of free form LEVA drug and (**C**) in vitro cumulative release profile of a suspension of LEVA/PVP/PMMA microparticles.

**Figure 5:**
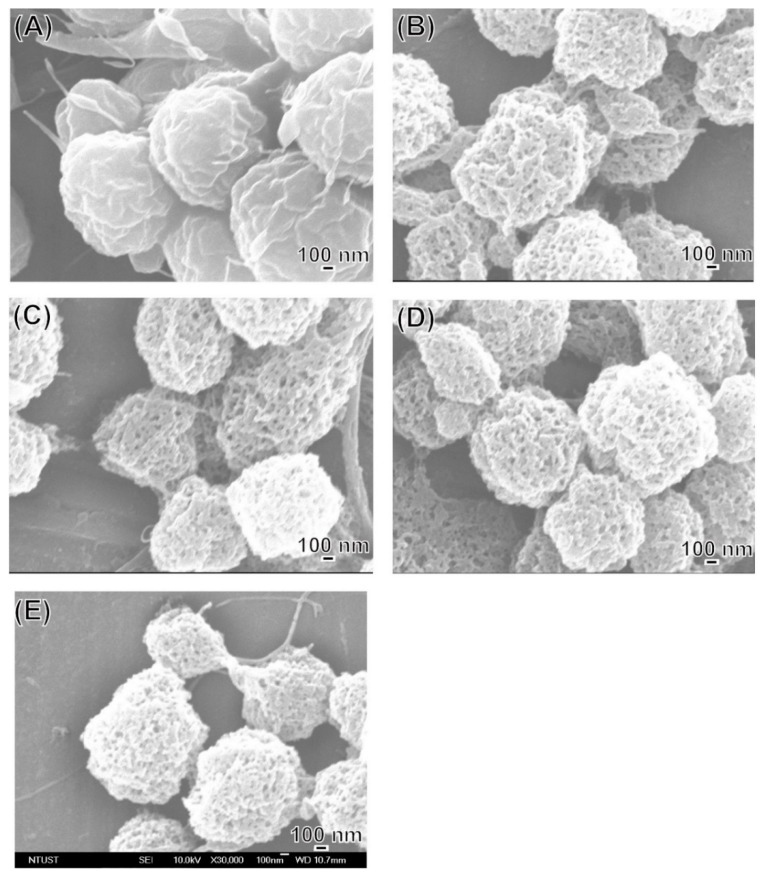
SEM images of LEVA/PVP/PMMA microparticles infiltrated in distilled water at the time points of (**A**) 0, (**B**) 20, (**C**) 40, (**D**) 90 min and (**E**) 24 h.

To investigate the LEVA release behavior of LEVA/PVP/PMMA MPs, the MPs were incubated in distilled water. First, UV–vis absorption spectroscopy was performed and the maximum absorption wavelength of free LEVA was determined to be 220 nm. Figure 4A illustrates a standard curve constructed from a series of LEVA solutions with known concentrations. This standard curve was set as the foundation for the subsequent estimation of the cumulative release of LEVA. Figure 4C shows an in vitro cumulative release profile of a suspension of LEVA/PVP/PMMA MPs compared with that of an aqueous solution of free LEVA (Figure 4B). At the timepoints of 1 and 24 h, the cumulative release of free LEVA was quantified to be approximately 50% and 90%, respectively, whereas that of LEVA encapsulated in the PVP/PMMA carriers was nearly 0.5% and 70%, respectively. To further understand the mechanism of drug release, Figure 5 illustrates SEM images of LEVA/PVP/PMMA MPs infiltrated in distilled water at the timepoints of 0, 20, 40, and 90 min and 24 h. At the beginning, the surfaces of the LEVA/PVP/PMMA MPs were smooth, but after soaking for 20 min in water, their surface developed numerous pores, attributable to erosion due to the hydrophilic nature of PVP. These results demonstrate that the relatively slow release rate was attributable to the tortuous polymeric diffusion pathways in the encapsulating matrix material and that this release rate could be accelerated after dissolving PVP in the medium.

### 3.3. Preparation of CD133-Conjugated LEVA/PVP/PMMA Microparticles

**Figure 6:**
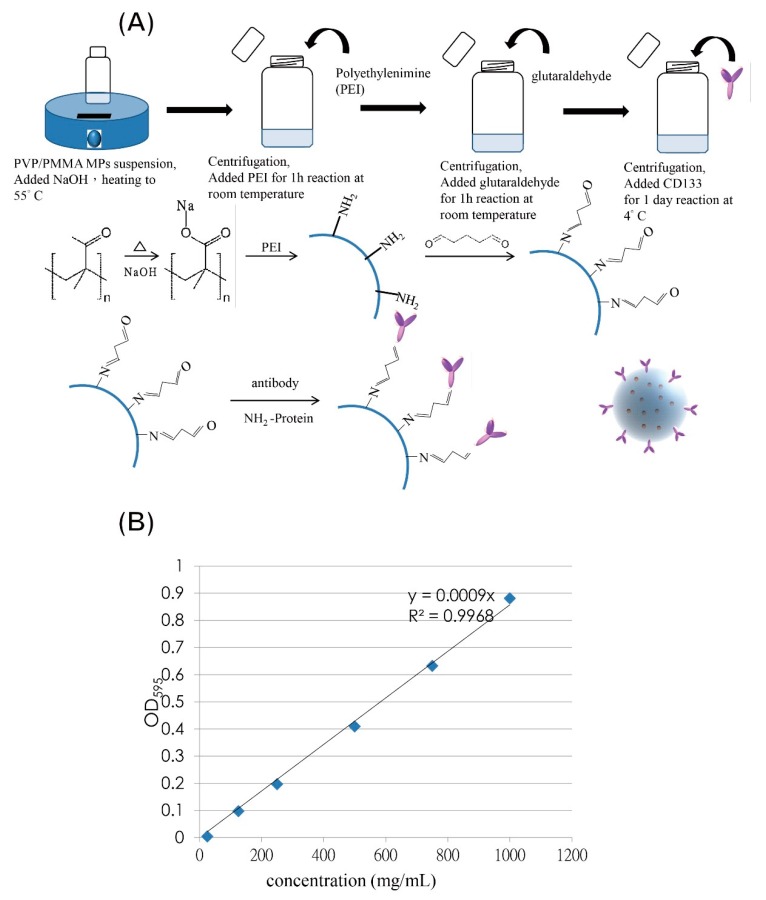
(**A**) Schematic illustration of facile protocol for synthesizing CD133-conjugated LEVA/PVP/PMMA microparticles. (**B**) Standard curve of Bradford assays established by using the BSA standard protein to determine the conjugation amount of antibody on the particle’s surface.

Figure 6A illustrates a facile protocol for synthesizing CD133-conjugated LEVA/PVP/PMMA microparticles. Firstly, the surface alkaline hydrolysis modification to generate a carboxylate group is necessary, as the PVP and PMMA polymers did not bear any functional groups for further bioconjugation with the PEI. Subsequently, a preformed CD133 antibody was added to the modified LEVA/PVP/PMMA microparticles’ particle suspension for antibody coupling at 4 °C. In this newly developed protocol, no laborious bioconjugation technique was needed and the ES technique could generate LEVA-encapsulated LEVA/PVP/PMMA microparticles within single step. Figure 6B shows the standard curve of Bradford assays established by using the BSA standard protein to determine the conjugation amount of antibody on the particle’s surface. Based on this analysis, we knew that each volume of particle suspension contained 821 µg/mL antibody conjugation.

### 3.4. Characterization of CD133-Conjugated LEVA/PVP/PMMA Microparticles

**Figure 7:**
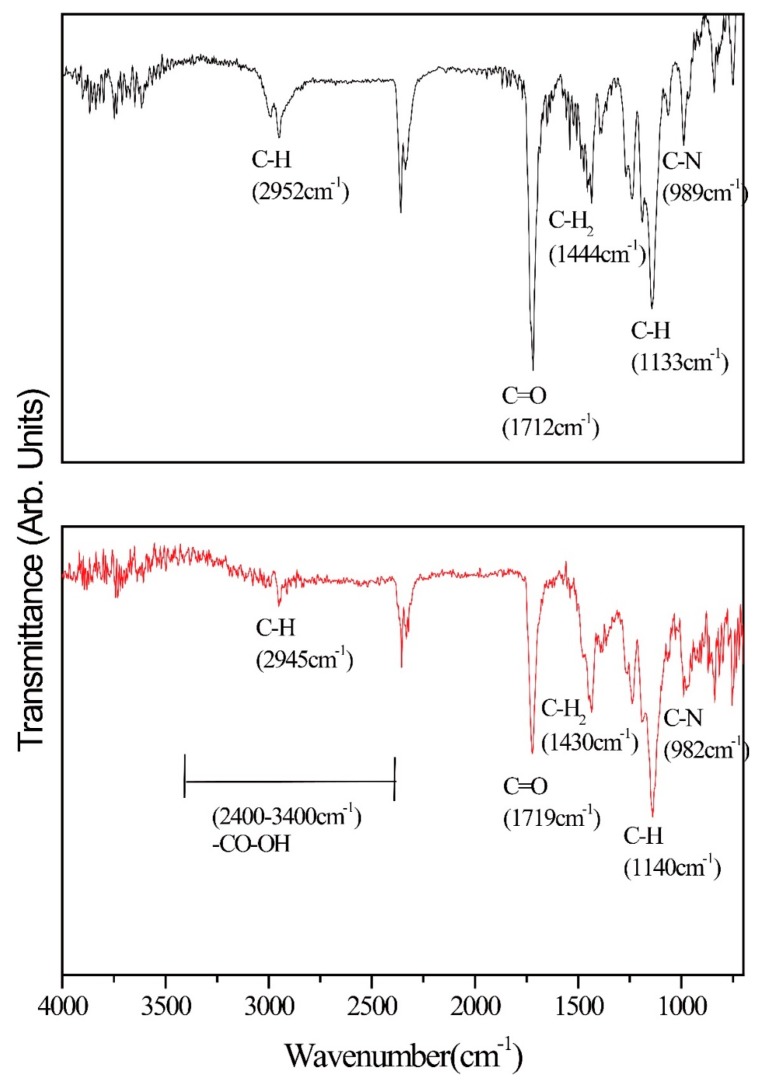
(**top**) FT-IR spectrum of LEVA/PVP/PMMA microparticles before hydrolysis. (**bottom**) FT-IR spectrum of LEVA/PVP/PMMA microparticles after hydrolysis.

**Figure 8:**
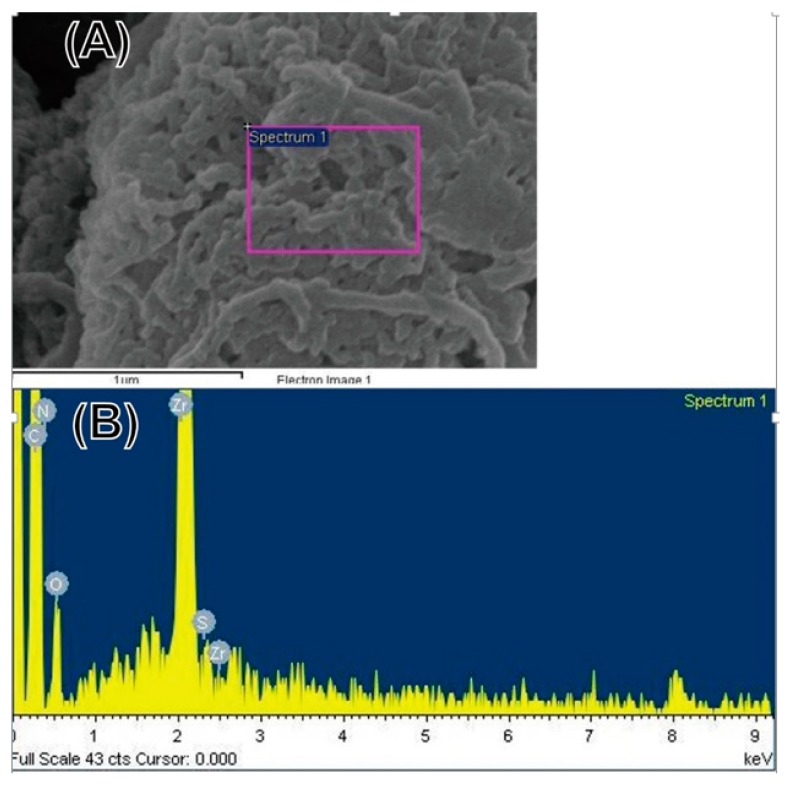
(**A**) SEM image of CD133-conjugated LEVA/PVP/PMMA MPs. (**B**) EDX spectrum acquired from the selected area on the CD133-conjugated LEVA/PVP/PMMA MPs.

Herein, the typical FT-IR equipped with an attenuated total reflection (ATR) accessory was used to characterize a series of pure LEVA/PVP/PMMA or CD133-conjugated LEVA/PVP/PMMA MPs. As shown in Figure 7, the data for LEVA/PVP/PMMA microparticles reveal two prominent peaks at 1710 cm^−1^ and 2952 cm^−1^, which can be attributed to the carbonyl group (-CO-) and saturated hydrocarbon (-CH_3_) group stretching mode in PMMA polymeric chain. On the contrary, when the LEVA/PVP/PMMA MPs were subject to sodium hydroxide alkaline hydrolysis, a typical feature of the stretching mode of the carboxylic acid group shows a broad band starting from 2400 cm^−1^ to 3400 cm^−1^ and thus significantly reduces the intensity of the saturated hydrocarbon (-CH_3_) group stretching mode at approximately 2945 cm^−1^, which is evidence of the formation of the carboxylic group after the hydrolysis step. This key procedure set up the foundation for the following CD133 antibody conjugation. Figure 8A shows the SEM image of individual CD133-conjugated LEVA/PVP/PMMA MPs. Obviously, the surface morphology reveals a layer of strip-like product covering on MPs. The selected area EDX spectrum (Figure 8B, Zr are attributed to the bottom substrate) shows the characteristic peaks of element C, N, O and S, which are all features of the antibody. The above results indicate the successful conjugation of antibody to the LEVA/PVP/PMMA MPs and these drug carriers can be used as a vehicle to selectively deliver therapeutic agent to the CD133 overexpressed tumor cells and make it easier for the uptake process by the lymphatic system, due to its big size.

### 3.5. Cytotoxicity of CD133-Antibody LEVA/PVP/PMMA MPs

**Figure 9:**
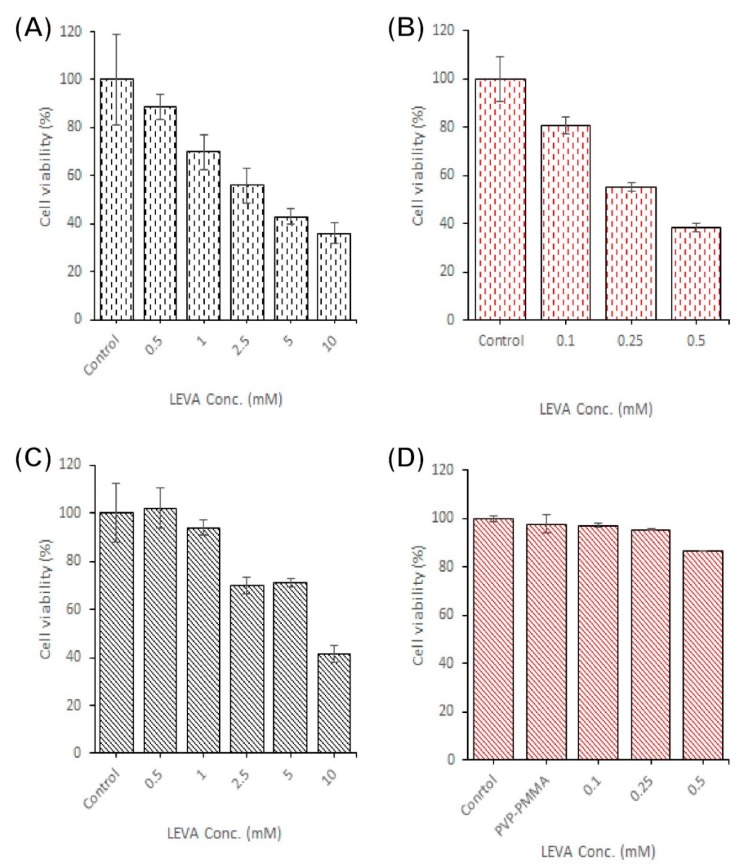
Cell viability of SKOV-3 and CP70 after being treated with a series of different concentration of (**A**,**B**) free form LEVA toward SKOV-3 and CP70, respectively. (**C**,**D**) CD133-conjugated Leva/PVP/PMMA MPs toward SKOV-3 and CP70, respectively. The results of quantitative analyses of the cytotoxicity of the aforementioned formulations against SKOV-3 and CP70 were determined by the MTT assays.

An MTT assay was employed to assess the cytotoxicity of a series of formulations with multiple LEVA concentrations against the ovarian cancer cell lines SKOV-3 and CP70. The formulations dispersed in PBS at multiple concentrations were (1) LEVA free-form solution and (2) CD133-conjugated LEVA/PVP/PMMA MPs. Figure 9 presents the results of quantitative analyses of the cytotoxicity of the these formulations against SKOV-3 and CP70 cells: The cells were treated for 72 h with multiple concentrations of LEVA formulations, namely 0.1–0.5 mM LEVA free-form solution (Figure 9A,C) and 0.5–10 mM for CD133-conjugated LEVA/PVP/PMMA MPs (Figure 9B,D). For SKOV-3 cells, treatment with free-form LEVA or CD133-conjugated LEVA/PVP/PMMA MPs exerted dose-dependent cytotoxic effects on SKOV-3 cell viability (Figure 9A,B). However, CD133-conjugated LEVA/PVP/PMMA MPs demonstrated no significant dose-dependent cytotoxic efficacy toward CP70 cells (Figure 9C,D). Based on our previous results [30] and references cited therein, both CD44 and CD133 receptors are overexpressed on SKOV-3 cells, at a rate approximately 1.47 times higher than that of CP70 cells. Moreover, CD133 receptors, highly expressed by ovarian and hematological CSCs, can lead to many cancer cells becoming resistant to current chemotherapeutic reagents. Thus, the CD133-conjugated LEVA/PVP/PMMA MPs are highly suitable for targeting CD133-overexpressed cancer cells or CSCs, possibly by inhibiting the alkaline phosphate activity [31].

## 4. Conclusions

Here, we developed novel LEVA/PVP/PMMA MPs using a single-step ES process. These MPs have an antitumor effect in the SKOV-3 and CP70 ovarian cancer cell lines and their slow release of LEVA. To the best of our knowledge, no studies on formulations of LEVA, PVP, and PMMA for ovarian cancer treatment have been conducted. Moreover, it can simultaneously be easily modified and turned to use as chemotherapy, hyperthermia therapy, and embolization at hepatic cancerous sites due to its large size, and thus is expected to exhibit greater antitumor efficacy than conventional therapy, but with mild side effects for the patient.
